# The Safety of Xenoestrogens

**Published:** 2004-11

**Authors:** Mary Eubanks

## Challenging the Genomic Model of Effects

Current thinking holds that environmental estrogens cause endocrine disruption when these steroid mimics enter the cell’s nucleus and turn genes on or off, or up or down, by binding to DNA. According to this genomic–nuclear pathway model, many xenoestrogens are viewed as harmless to humans and wildlife because exposure to high levels of chemical is necessary before there is a change in gene expression. However, the genomic model assumes a long, multistep process of macromolecular synthesis; it does not fully account for empirical evidence that the signal response to some hormones is so fast it must be initiated outside the cell via membrane receptors connected to fast-acting molecules. An alternative membrane-initiated hypothesis is just beginning to be addressed and tested. This month, Nataliya N. Bulayeva and Cheryl S. Watson of the University of Texas Medical Branch report experimental evidence that challenges the prevailing genomic paradigm of endocrine disruption **[EHP** **112:1481–1487]**.

For their experiments, the scientists employed a prolactinoma cell line derived from a rat pituitary gland that has been a model experimental cell line for over 30 years. A subline of these cells that exhibits fast responses and is sensitive to estrogens at small doses provides a good test system for the study of nongenomic responses to estrogenic compounds. This system allows researchers to investigate questions about mechanisms by measuring functional end points of estrogen action, such as prolactin secretion, which increases upon exposure to estrogen.

Extracellular signal–regulated protein kinases, or ERKs, belong to a large class of enzymes involved in cell signaling pathways that generate signals to multiple end points. They are good indicators of non-genomic estrogenic activity because ERK activation is mediated by phosphorylation, a signal from outside the cell. When ERKs are activated by exposure to estrogens or compounds that mimic estrogens, the cell’s medium turns yellow. The yellow product, which correlates with the amount of phosphorylated ERK identified by an antibody, can be measured so precisely that small changes in levels of phosphorylated ERK can be detected.

Bulayeva and Watson compared ERK activation by the most potent endogenous estrogen—estradiol—with activation by three major classes of xenoestrogens: organochlorine pesticides (endosulfan, dieldrin, and DDE, a DDT metabolite), detergents used in plastics manufacturing (*p*-nonylphenol and bisphenol A), and coumestrol (a phytoestrogen present in alfalfa sprouts, soybeans, and sunflower seeds/oil). The responses were measured at different concentrations over a 3- to 30-minute time course. Affected points along the activation pathway were subsequently investigated by the addition of specific inhibitors to each pathway participant.

The results showed that every xenoestrogen tested, except bisphenol A, exhibited strong ERK activation. Unexpectedly, individual compounds produced the effect at different times and concentrations specific to the particular compounds. Also, individual compounds were found to trigger specific pathways within the nongenomic signaling network leading to different end points. Coumestrol, endosulfan, and *p*-nonylphenol had an effect at extremely low picomolar levels, as low as estradiol. The authors concluded, “These very low effective doses for xenoestrogens demonstrate that many environmental contamination levels previously thought to be subtoxic may very well exert significant signal- and endocrine-disruptive effects, discernable only when the appropriate mechanism is assayed.”

This study sheds new light on the conundrum of why exposure to concentrations of environmental estrogens deemed safe by the genomic model exhibit well-documented harmful effects on wildlife. It raises new concerns about the effect of xenoestrogens on human health and important questions about the adequacy of current environmental protection policy and regulations based on the genomic model.

## Figures and Tables

**Figure:**
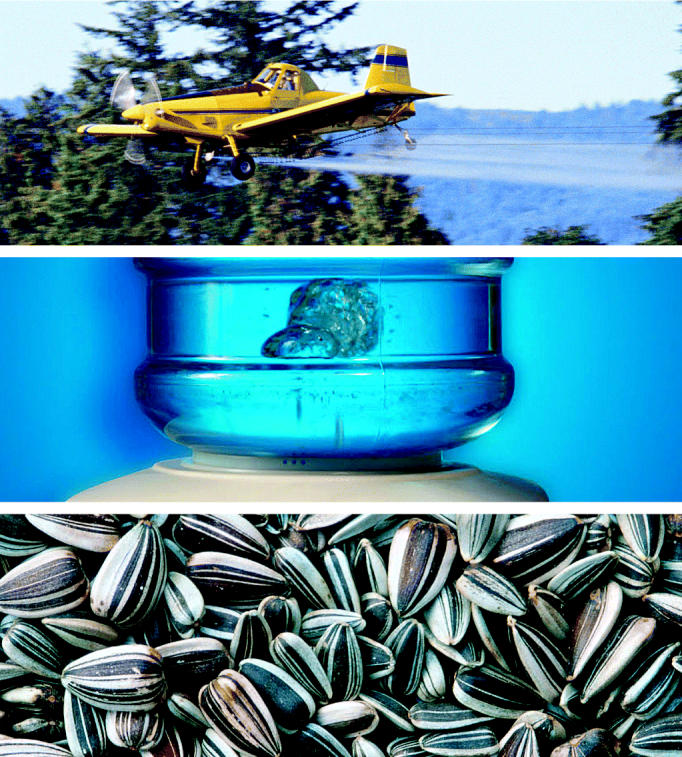
**New model explains differences.** Researchers compared ERK activation by three classes of environmental estrogens found in pesticides, plastics, and plants to that of estradiol. Results showed that, unlike previously believed, not all xenoestrogens act the same.

